# A Line Full of Lessons: A “Sake Lees”-Like Mass Revealing Bacillus cereus Contamination in an Amino Acid Infusion

**DOI:** 10.7759/cureus.100819

**Published:** 2026-01-05

**Authors:** Hajime Tsuboi, Shu Toda, Fumio Iwane

**Affiliations:** 1 General Internal Medicine, Kyoto Okamoto Memorial Hospital, Kyoto, JPN; 2 Cardiovascular Medicine, Kyoto Okamoto Memorial Hospital, Kyoto, JPN; 3 Laboratory Medicine, Kyoto Okamoto Memorial Hospital, Kyoto, JPN

**Keywords:** bacillus cereus, catheter-related bloodstream infection, infection control, infusion line contamination, parenteral nutrition

## Abstract

*Bacillus cereus* (*B. cereus*) is a known cause of catheter-related bloodstream infections (CRBSIs) in healthcare settings, with increased risk observed in immunocompromised patients and those receiving amino acid-based parenteral nutrition. This condition is typically identified by local signs at the catheter insertion site and positive blood cultures. We report a case of an elderly hospitalized patient in whom an unusual visual finding, whitish, turbid, “sake-lees”-like crystalline aggregates within the infusion line, contributed to the diagnosis of *B. cereus*-associated CRBSIs. This case underscores the potential routes of *B. cereus *contamination in the medical environment and highlights the importance of rigorous infection control practices during infusion preparation and catheter-related procedures for hospitalized patients.

## Introduction

*Bacillus cereus* (*B. cereus*) is a spore-forming, Gram-positive bacillus that is widely distributed in nature, including soil and aquatic environments, and is also recognized as part of the normal intestinal flora in healthy adults [1]. Human infections typically occur via oral or contact transmission. Orally transmitted infections are often associated with food poisoning outbreaks [2], whereas contact transmission is mainly observed in healthcare settings, with contaminated linens and environmental surfaces serving as sources of infection [3].

*B. cereus* is also a known causative organism of catheter-related bloodstream infections (CRBSIs). Although *B. cereus *isolated from blood cultures is often regarded as a contaminant, it can represent true bacteremia, particularly in immunocompromised patients; in such individuals, CRBSIs are a common source of infection [4]. Additionally, patients receiving amino acid-based parenteral nutrition are known to be at increased risk of developing *B. cereus*-related CRBSIs [5]. In general, *B. cereus*-related CRBSIs are identified either through skin findings such as erythema or swelling at the catheter insertion site or by the detection of the organism in blood cultures.

In contrast, the present case was characterized by a unique visual finding: the infusion line was filled with a whitish, turbid, sake-lees-like crystalline mass in the absence of skin changes at the catheter insertion site. This finding provided valuable insights into the route of *B. cereus* contamination and highlighted the critical importance of infection control measures against environmental and device-associated infections in healthcare settings.

## Case presentation

An 89-year-old man with a medical history of gastric antral vascular ectasia, aortic stenosis, and Heyde’s syndrome was admitted with anorexia and general fatigue. The patient was diagnosed with aspiration pneumonia. Antimicrobial therapy with ceftriaxone was initiated. By hospital day seven, the pneumonia had improved; however, the patient’s swallowing function, which had already been declining, had deteriorated to the point of near abolition. A nasogastric tube was inserted, and enteral nutrition was started. At the same time, peripheral intravenous administration of an amino acid infusion was also initiated to supplement his nutrition, as his caloric intake via enteral nutrition was insufficient due to persistent hypoalbuminemia and a tendency toward diarrhea.

On hospital day 11, the patient developed a fever accompanied by chills and rigors. Erythema and swelling were noted at the insertion site of the peripheral catheter used for amino acid infusion, raising suspicion of CRBSI. The next day, both sets of previously collected blood cultures tested positive for *B. cereus*, and intravenous vancomycin (VCM) therapy was initiated. The peripheral venous line, which was suspected to be the source of infection, was promptly removed and replaced with a non-tunneled central venous catheter (CVC) inserted via the right internal jugular vein. The patient’s fever and inflammatory markers gradually improved, and VCM therapy was discontinued on hospital day 21. During the subsequent week, the patient remained afebrile and clinically stable, although his nutritional status did not significantly improve.

**Figure 1:**
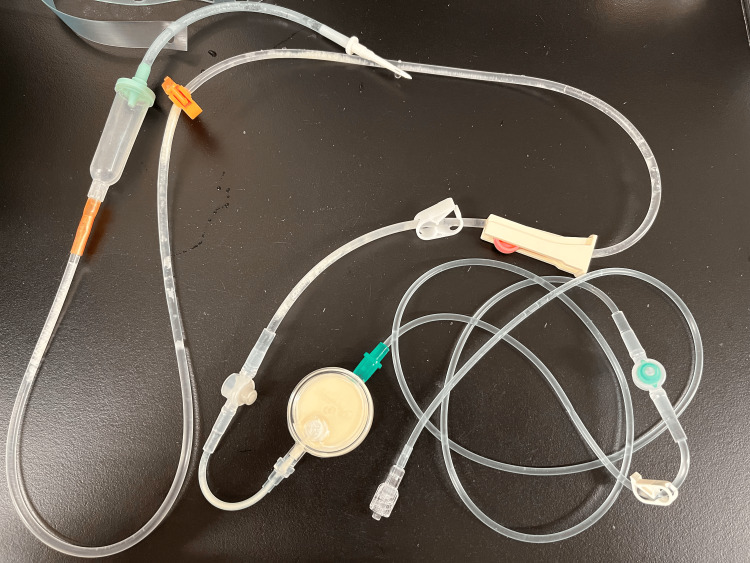
Appearance of the central venous infusion line used for amino acid solution administration. The lumen is filled with a white, cloudy, “sake lees”-like crystalline mass extending from the infusion port. The distribution of this crystalline material, accumulated predominantly on the infusion pack side, proximal to the inline filter, while the lumen beyond the filter toward the patient remains clear, suggests that *B. cereus* entered through the infusion port rather than via retrograde contamination from the patient or insertion site. This distinctive finding highlights the potential for external contamination of amino acid preparations in patients with *B. cereus* bacteremia and underscores the importance of strict infection control measures during line manipulation.

On hospital day 28, the infusion line was replaced. However, on day 30, the patient again developed a fever exceeding 38°C, along with elevated inflammatory markers on blood tests. That evening, a nurse noticed that the CVC line used for amino acid infusion was filled with a whitish, turbid, “sake-lees”-like crystalline mass (Figure 1).

**Figure 2:**
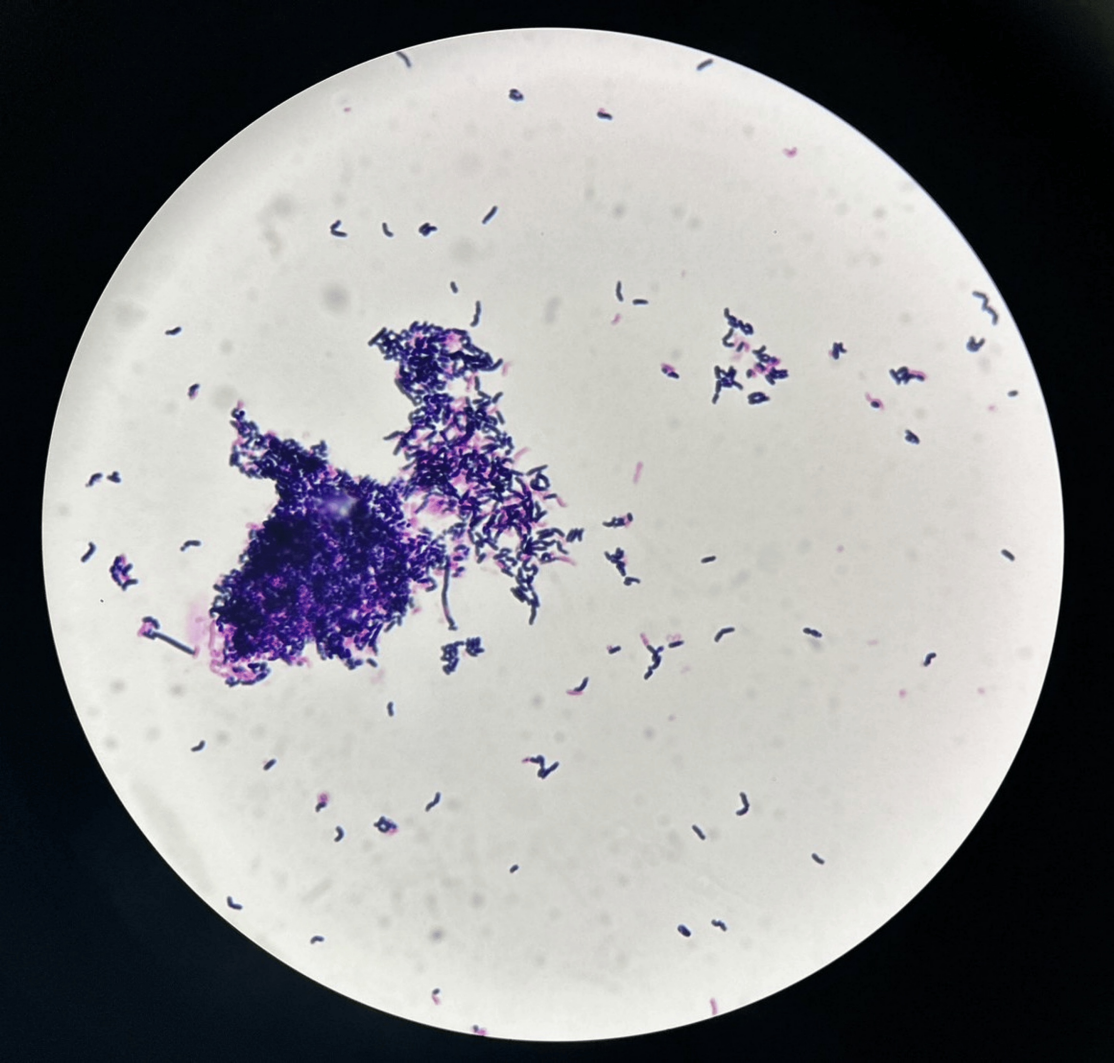
Gram stain of the white sediment extracted from the infusion line. Gram staining of the “sake lees”-like crystals collected from the infusion route revealed numerous Gram-positive bacilli consistent with the genus *Bacillus*. Dense bacterial aggregation was observed in the central region.

The line was promptly removed, and Gram staining of the intraluminal deposits revealed Gram-positive, rod-shaped bacterial aggregates consistent in morphology with *B. cereus* (Figure 2).

Subsequent cultures of both the crystalline material and concurrently collected blood samples confirmed the presence of *B. cereus*. The antimicrobial susceptibility profiles of the isolates from both the first and second episodes were identical; the results are summarized in Table 1.

**Table 1:** Antimicrobial susceptibility profiles of the Bacillus cereus isolates. CLSI: Clinical and Laboratory Standards Institute; MIC: minimum inhibitory concentration

Antimicrobial agent	MIC (µg/mL)	CLSI interpretation
Penicillin	8	R
Ampicillin/Sulbactam	2	R
Cefazolin	≧32	R
Meropenem	≦0.5	S
Clindamycin	1	I
Vancomycin	1	S
Levofloxacin	≦1	S
Sulfamethoxazole-trimethoprim	≧152	R

The hospital’s infection control team (ICT) intervened and, based on the distribution of the crystalline material within the infusion line, suspected contamination during preparation or at the time of piercing the infusion port. This prompted re-education and reinforcement of aseptic techniques during infusion preparation and handling. Additionally, the ICT disseminated information about the case hospital-wide, prompting all departments to review infection control measures related to infusion handling. Intravenous VCM therapy was resumed, and defervescence along with improvement in inflammatory markers was subsequently observed.

## Discussion

We experienced a case of CRBSI caused by *B. cereus*, presenting with a distinctive visual finding: a sake-lees-like crystalline mass within the infusion line. While *B. cereus* is a known pathogen in nosocomial infections and CRBSIs, to our knowledge, reports visually capturing intraluminal colonization of the catheter by this organism are exceedingly rare.

*B. cereus*-associated CRBSIs have been linked to the use of amino acid-based parenteral nutrition [5]. Additional reported risk factors for *B. cereus *bacteremia include immunosuppression, the use of CVCs, valvular heart disease (particularly post-valve replacement), prior antibiotic therapy, non-oral nutritional support, and hematologic malignancies [5-7]. Based on his comorbidities and inpatient clinical course, the present patient exhibited multiple risk factors, including advanced age (representing impaired immunity), CVC use, non-oral nutrition, and a history of valvular disease.

Typically, *B. cereus*-related CRBSIs are identified either through local skin findings (such as erythema or swelling at the catheter insertion site) or via positive blood cultures. Visual confirmation of bacterial aggregates within the infusion line, as in our case, is extremely rare. CRBSIs may occur without overt skin findings at the insertion site [8], rendering diagnosis highly dependent on blood cultures and raising the risk of missed or delayed detection. In this case, the sake-lees-like crystalline mass was observed during the patient’s second episode of *B. cereus* bacteremia within the same hospitalization. Unlike the first episode, no local signs of infection were noted on physical examination. The only clinically evident findings were the sudden onset of fever and elevated inflammatory markers. Given the absence of local signs at the time of detection, it is likely that the visualized crystalline mass represented the earliest observable manifestation of infection. Furthermore, this unique “sake-lees” appearance provides a striking example of rapid bacterial colonization. This phenomenon may be attributed to the biofilm-forming characteristics of* B. cereus*, which are known to be enhanced in the presence of amino acid solutions, facilitating such macroscopic aggregations [9]. Therefore, our experience suggests that inpatients with risk factors for *B. cereus *bacteremia who develop fever or elevated inflammatory markers should be considered for blood culture testing at a lower threshold.

The crystalline material was localized predominantly in the segment of the infusion line extending from the amino acid bag to the filter. In contrast, the downstream segment (toward the patient) remained macroscopically clear. This distribution suggests that* B. cereus* likely entered through the infusion port rather than via the patient’s bloodstream, insertion site, or CVC connection. Contamination may have occurred during infusion preparation or disinfection and puncture of the port.

In retrospect, the patient’s multiple risk factors, along with a prior episode of* B. cereus* bacteremia, indicating possible environmental colonization (e.g., skin or linens), should have prompted stricter adherence to disinfection protocols during line handling and infusion bag exchange. In addition, to further reduce the risk of CRBSI caused by *B. cereus*, earlier discontinuation of parenteral nutrition and prompt catheter removal could have been considered once enteral nutrition via a nasogastric tube had been established.

Following this recurrent episode, our hospital’s ICT implemented reinforced education on aseptic techniques during infusion preparation and handling. Although no horizontal transmission was detected, several previous reports have documented contact transmission of *B. cereus* via contaminated healthcare environments [10,11]. Thus, strict adherence to personal protective equipment protocols remains critical to preventing nosocomial spread.

## Conclusions

This case highlights the importance of rigorous infection control measures, particularly during amino acid infusion preparation, port puncture, and all catheter-related procedures, for patients at risk of *B. cereus* bacteremia, including those with a prior infection history.

## References

[1] Bottone EJ (2010). Bacillus cereus, a volatile human pathogen. Clin Microbiol Rev.

[2] Dietrich R, Jessberger N, Ehling-Schulz M, Märtlbauer E, Granum PE (2021). The food poisoning toxins of Bacillus cereus. Toxins (Basel).

[3] Sasahara T, Hayashi S, Morisawa Y, Sakihama T, Yoshimura A, Hirai Y (2011). Bacillus cereus bacteremia outbreak due to contaminated hospital linens. Eur J Clin Microbiol Infect Dis.

[4] Funada H, Uotani C, Machi T, Matsuda T, Nonomura A (1988). Bacillus cereus bacteremia in an adult with acute leukemia. Jpn J Clin Oncol.

[5] Kutsuna S, Hayakawa K, Kita K (2017). Risk factors of catheter-related bloodstream infection caused by Bacillus cereus: case-control study in 8 teaching hospitals in Japan. Am J Infect Control.

[6] Shimada T, Ishikawa K, Kawai F, Yoneoka D, Mori N (2023). Risk factors associated with infection-related mortality of Bacillus cereus bacteremia in hematologic disorders. Int J Hematol.

[7] Gopinathan A, Kumar A, Sen AC (2018). A case series and review of Bacillus cereus endocarditis from India. Open Microbiol J.

[8] Buetti N, Ruckly S, Lucet JC (2020). Local signs at insertion site and catheter-related bloodstream infections: an observational post hoc analysis using individual data of four RCTs. Crit Care.

[9] Hsueh YH, Somers EB, Wong AC (2008). Characterization of the codY gene and its influence on biofilm formation in Bacillus cereus. Arch Microbiol.

[10] Glasset B, Herbin S, Granier SA (2018). Bacillus cereus, a serious cause of nosocomial infections: epidemiologic and genetic survey. PLoS One.

[11] Yamada K, Shigemi H, Suzuki K, Yasutomi M, Iwasaki H, Ohshima Y (2019). Successful management of a Bacillus cereus catheter-related bloodstream infection outbreak in the pediatric ward of our facility. J Infect Chemother.

